# Editorial: Food safety and public health

**DOI:** 10.3389/fmicb.2023.1169139

**Published:** 2023-03-09

**Authors:** Yosra A. Helmy, Hosny El-Adawy, Yasser M. Sanad, Mostafa Ghanem

**Affiliations:** ^1^Department of Veterinary Science, College of Agriculture, Food and Environment, University of Kentucky, Lexington, KY, United States; ^2^Institute of Bacterial Infections and Zoonoses, Friedrich Loeffler Institut, Jena, Germany; ^3^Faculty of Veterinary Medicine, Kafrelsheikh University, Kafr El-Sheikh, Egypt; ^4^Department of Agriculture, University of Arkansas, Pine Bluff, AR, United States; ^5^Department of Epidemiology, University of Arkansas for Medical Sciences, Little Rock, AR, United States; ^6^Department of Veterinary Medicine, University of Maryland, College Park, MD, United States; ^7^Virginia-Maryland College of Veterinary Medicine, College Park, MD, United States

**Keywords:** food safety, public health, foodborne, antimicrobial resistance (AMR), bacteria

## Introduction

Foodborne illnesses have a major impact on food safety and public health worldwide. In the USA alone, foodborne illnesses lead to ~128,000 hospitalizations and around 3,000 deaths yearly, based on Centers for Disease Control and Prevention (CDC) estimates. They cost billions of dollars in healthcare-related and industry per year. Currently, the control of foodborne pathogens depends mainly on preventing the introduction and spread of infectious agents and on using antibiotics. The number of antibiotic-resistant bacteria isolated from humans and animals has increased over the last two decades due to the misuse of antibiotics in both human and food-producing animals, leading to the global pandemic of antibiotic-resistant bacteria. In addition, infection of humans with antibiotic-resistant bacteria leads to an increased incidence of treatment failure and disease severity, negatively affecting public health. Therefore, there is a critical need to proactively devise alternative approaches to control foodborne pathogens that can help in reducing the burden of foodborne bacterial pathogens and antimicrobial resistance (AMR) on public health and the economy. The main aim of this editorial is to provide a comprehensive update on the efforts to control and mitigate the negative impact of foodborne pathogens and associated antibiotic resistance on humans, animals, food, and the environment. We have received an excellent collection of articles (32 articles) that cover multiple aspects including but not limited to (1) understanding the epidemiology of foodborne pathogens, (2) characterization of ecology AMR in food supply chain, (3) development of antibiotic-independent and cost-effective approaches for disinfection of food, and (4) development of improved diagnostic methods for detection of foodborne pathogens. The common themes of these articles are adopting the One Health approach to tackle foodborne pathogens and AMR at the animal–human–environment interface and using genomic approaches to investigate the foodborne outbreaks and understand the ecology of AMR. Here, we provide a brief commentary on some of the work that has been presented under different aspects outlined in our Research Topic.

## Understanding the epidemiology of foodborne pathogens and ecology antimicrobial resistance in food supply chain

Kubicová et al. reported the genomic diversity of *Listeria monocytogenes* isolates from slovakia (2010–2020). Listeriosis is a serious public health concern, particularly in European countries like Slovakia. That study, which analyzed 988 *L. monocytogenes* isolates from the 2010–2020, provides important insights into the population genetic structure of *L. monocytogenes* in Slovakia and highlights the need for continued monitoring of Listeriosis in food products to improve the understanding of the circulation of this pathogen and to ensure public health protection.

Suwono et al. investigated the regional associations of AMR *E. coli* isolated from human and food-producing animal in three different regions in Germany. The results showed that human isolates from different health care facilities tended to cluster together regardless of the regions of origin, with a slightly stronger association observed for the level of health care as compared to the regional stratification. On the other hand, Closer regional associations were seen for some of the food-producing animal populations. This study highlights the importance of understanding the potential associations between different populations of AMR bacteria in order to improve the understanding of the circulation of these bacteria and imply public health and food safety measures.

Gan et al. investigated the genomic landscape and phenotypes of *Cronobacter sakazakii* isolated from raw material, environment, and production facilities in powdered infant formula factories in china. *Cronobacter* contamination in powdered infant formula (PIF) and infant foods is a significant public health concern, as it can cause severe infections in neonates, particularly in low-birth-weight neonates. In that study, 15 *Cronobacter* strains were isolated from raw materials, production facilities, and the environment of two PIF factories using conventional bacteriological methods and whole-genome sequencing (WGS). All isolates were identified as *C. sakazakii*, and the detection of *C. sakazakii* from different PIF production sources indicated that this bacterium could disseminate through raw ingredients, along with soil cross-contamination in the PIF production site. The authors suggested that PIF manufacturing factories should improve supervision of the environment around the factory and strengthen the hygiene management of raw materials. They also highlighted the strategies to prevent the persistence of *C. sakazakii* in the PIF factory, including limiting the contamination of processing facility's surfaces.

Biggel et al. investigated the potential link between 13 biopesticide isolates and 20 *Bacillus thuringiensis* isolates from food and human stool. The use of biopesticides such as *B. thuringiensis* is becoming increasingly popular in agriculture to control pests. This study provided strong evidence that biopesticides are the source of *B. thuringiensis* on food products and raises concerns about the potential health risks to consumers. It is important for regulatory agencies to closely monitor the use of biopesticides in the future to understand the potential health risks of *B. thuringiensis*.

Rocha et al. performed a meta-analysis study to understand the dynamics of *Salmonella* in environmental water sources. Given the importance of water quality for agri-food systems and the public health significance of *Salmonella*, it is crucial to better understand these dynamics to develop more effective strategies to control salmonellosis. The authors suggested that conducting longitudinal study and serotyping can help to understand seasonal variations and the effect of other factors. Additionally, high-throughput approaches like metagenomics can provide valuable information about complex relationships between *Salmonella* and other biotic factors. Overall, this meta-analysis highlights the need for more comprehensive and detailed studies to fully understand the prevalence and distribution of *Salmonella* in water and how this relates to public health. Identifying the key factors that can affect the recovery rate and serovar representation of *Salmonella* in water will be essential to develop effective strategies to control and prevent salmonellosis.

Ahmed and Gulhan summarized the Campylobacteriosis status in wild birds and the possibility of interspecies transmission. In a comprehensive review, the authors showed that *Campylobacter* has been isolated from various species of wild birds worldwide, with *C. jejuni* being the most isolated species. The prevalence of *Campylobacter* in wild birds is associated with geographical location, season, the bird's health status, bird species, sample type, the method used, and ecological factors. In this review, the authors emphasize that wild birds carry strains of *Campylobacter*, which are indistinguishable from domestic animals and humans and are therefore an important public and animal health concern. The role of wild birds in the epidemiology of *Campylobacter* should not be undermined as drug-resistant strains, especially resistance to tetracycline and fluoroquinolones, are increasingly documented. Detail-oriented epidemiological investigations characterizing the genetic relatedness of isolates from the respective species and environment through one health approach are warranted.

Liu et al. investigate the prevalence, antimicrobial susceptibility, and virulence genes of *S. aureus* in 125 raw milk samples collected from 50 goats, 25 buffalo, 25 camels and 25 yaks collected from 5 provinces in China in 2016. Thirty-six *S. aureus* were isolated, 26 strains (26/36, 72.2%) showed antibiotics resistance, and six strains isolated from goats were identified as methicillin resistant *S. aureus* (MRSA). Nineteen *S. aureus* (52.8%) were considered as multidrug resistant. The resistance genes were detected in 25 *S. aureus*. The most predominant resistance genes were *bla*Z (69.2%), *aac*60-*aph*200 (50.0%), and *tet*(M) (38.5%). The *mec*A, *ant* (6)-Ia and *fex*A gene were only detected in *S. aureus* from goat milk. The authors' findings indicated that the prevalence and antimicrobial resistance of *S. aureus* was a serious concern in different raw milks in China, especially goat milks.

Parra-Flores et al. used whole-genome sequencing (WGS) to test *in vitro* and *in silico Listeria monocytogenes* strains isolated from ready-to-eat (RTE) foods for virulence factors and antibiotic resistance. RTE foods are the most common route of transmission for the pathogen. In RTE food samples, the overall positivity of *L. monocytogenes* was 3.1%, and 14 strains were isolated. Average nucleotide identity, ribosomal multilocus sequence typing (rMLST), and core genome MLST were used to identify L. monocytogenes ST8, ST2763, ST1, ST3, ST5, ST7, ST9, ST14, and ST451 sequence types. There were seven isolates with serotypes 1/2a, 1/2b, 4b, and 1/2c. Three strains were found to be resistant to ampicillin *in vitro*, and all of them carried the resistance genes fosX, lin, norB, mprF, tetA, and tetC. Additionally, the genes arsBC, bcrBC, and clpL, which conferred resistance to stress and disinfectants, were discovered. Almost thirty-two of the strains displayed the bsh, clpCEP, hly, hpt, iap/cwhA, inlA, inlB, ipeA, lspA, mpl, plcA, pclB, oat, pdgA, and prfA genes. All strains also contained the prfA and hlyA genes. A premature stop codon (PMSC) of type 11 was found in one isolate's inlA gene, while a new mutation (deletion of A at position 819) was found in another isolate. Nine isolates included MGEs and the plasmids Inc18(rep25), Inc18(rep26), and N1011A. CAS-Type II-B systems were observed in ten isolates; In addition, three genomes contained Anti-CRISPR AcrIIA1 and AcrIIA3 phage-associated systems. The strains isolated from the RTE foods have traits of virulence and antibiotic resistance, pointing to a potential threat to consumers' public health.

## Development of antibiotic-independent approaches for improve food safety

Jiang, Xin et al. reported the discovery of LFX01, a novel bacteriocin found in fish intestines that can inhibit the growth of *Shigella flexneri*. That study highlights the potential of LFX01 as a novel and effective antibacterial agent in the food industry, with its excellent acid-base and temperature tolerance. The study helps to better understand the dynamics of foodborne pathogens in water, which can aid in the development of more effective strategies to control and mitigate salmonellosis.

Shen et al. investigated the effectiveness of gaseous ozone in reducing *Listeria innocua* and quality attributes and disorders of Red Delicious apples during long-term commercial cold storage. Overall, this study suggests that gaseous ozone application during controlled atmosphere (CA) storage is a viable strategy for controlling *Listeria* on fresh apples without negatively influencing apple quality attributes. However, more research is needed to understand the differences in behavior of *L. innocua* on different apple varieties and how storage conditions affect the overall microbial population.

Mariita et al. evaluated the efficacy of portable UVC devices, specifically the purgaty One system, in disinfecting bacterial contamination in static water. This study's results suggest that portable UVC devices could be useful in environments where people are vulnerable to pathogens, including travel medicine, healthcare facilities, hiking, remote military installations, and regions with water potability challenges.

Wang, Wang et al. investigated the detailed regulatory roles of Aa*VeA* in *Alternaria alternata* with various light sources from the comparative analyses between the wild type and the gene knockout strains. *A. alternata* is the most common species contaminating a wide range of plants which produces a variety of mycotoxins treating human and animal health. From differentially expressed genes (DEG) expression and further verification by RT-qPCR, the loss of AaVeA caused the discontinuous supply of the substrates for mycotoxin biosynthesis and the drastic decline of biosynthetic gene expression. In addition, pathogenicity depends on Aa*VeA* regulation in tomatoes infected by *A. alternata in vivo*. These findings provide a distinct understanding of the roles of Aa*VeA* in fungal growth, development, mycotoxin biosynthesis, and pathogenicity in response to various light sources.

Bland et al. have examined the potential effect of commercial Quaternary Ammonium Compound sanitizer on developing cross-resistance to select antibiotics in *Listeria monocytogenes* isolated from fresh produce environments. In this study six *L. monocytogenes* isolated from fresh produce handling, processing, and packing facilities were examined for their potential to develop cross-resistance to antibiotics and a commercial sanitizer. In the presence of reserpine, a well-known efflux pump inhibitor, all adapted (qAD) isolates regained their cQAC susceptibility. All tested isolates showed decreased sensitivity to chloramphenicol, ciprofloxacin, clindamycin, kanamycin, novobiocin, penicillin, and streptomycin. Ampicillin and gentamicin, two antibiotics commonly used to treat listeriosis, remained effective against qAD isolates. Comparative genomic analysis and whole genome sequencing of qAD strains revealed several mutations in fepR, the regulator for the FepA fluoroquinolone efflux pump. Following low-level adaptation to cQAC, the findings suggest that antibiotic susceptibility decreases because of fepR mutations. To better comprehend the likelihood of cross-resistance development in food chain isolates and its implications for the food industry, additional research into the cross-resistance mechanisms and pressures among *L. monocytogenes* isolates recovered from various sources is required.

Soltani et al. evaluated the gastrointestinal stability and activity of microcin J25, pediocin PA-1, bactofencin A and nisin using *in vitro* models. In addition, cytotoxicity, and hemolytic activity of these bacteriocins were investigated on human epithelial Caco-2 and rat erythrocytes, respectively. Pediocin PA-1, bactofencin A, and nisin were observed to lose their stability while passing through the gastrointestinal tract, while microcin J25 is only partially degraded. Besides, selected bacteriocins were not toxic to Caco-2 cells, and the integrity of the cell membrane was observed to remain unaffected in presence of these bacteriocins at concentrations up to 400 μg/mL. In hemolysis study, pediocin PA-1, bactofencin A, and nisin were observed to lyse rat erythrocytes at concentrations higher than 50 μg/mL, while microcin J25 showed no effect on these cells. According to data indicating gastrointestinal degradation and the absence of toxicity of pediocin PA-1, bactofencin A, and microcin J25 they could potentially be used in food or clinical applications.

## Development of improved diagnostic methods for detection and of foodborne pathogens

Wang, Ye et al. highlighted the need for more accurate and efficient methods for detecting *Pseudomonas aeruginosa* in food. The authors suggested that genotype-based identification methods such as PCR and qPCR, may be a promising approach for detecting *P. aeruginosa* in food and preventing food-related illness. However, further research is needed to evaluate the effectiveness of these methods and to explore new methods that may be more effective in detecting *P. aeruginosa* in food.

Tsai et al. have proposed Source-specific Bacteroides microbial source tracking (MST) markers as alternative indicators in animal-derived foods like milk products collected from vendors in urban Kenyan communities and infant foods made with the milk (*n* = 394 pairs). The samples were tested using conventional culture methods and TaqMan qPCR for enteric pathogens and human and bovine-sourced MST markers. The authors proved that MST markers were more frequently detected in infant food prepared by caregivers, indicating recent contamination events were more likely to occur during food preparation at home. However, Bacteroides MST markers had lower sensitivity in detecting enteric pathogens in food than traditional Enterobacteriaceae indicators. However, Bacteroides MST markers t could provide valuable information about how foods become contaminated; they may not be suitable for predicting the origin of the enteric pathogen contamination sources.

Jiang, Yang et al. used multiplex PCRs for rapid diagnosis of suspected cases in the lab; epidemiological evidence indicated that all patients had consumed egg sandwiches served as snacks to children and staff at a nursery in Dongguan, near Shenzhen. These analyses were complemented by near real-time multicenter whole-genome analyses that were completed within 34 h. Case patients, food handlers, kitchenware, and sandwiches with mayonnaise made in the kitchen all contained *Salmonella* Enteritidis. For outbreak-associated isolates, a well-supported cluster with pairwise distances between genomes of 1 single-nucleotide polymorphism (SNP) was found through whole-genome SNP analysis, establishing the definitive link between all samples. The minimum pairwise distance was >14 SNPs when compared to previous isolates from the same region, indicating a non-local outbreak source. A *S*. Enteritidis clone's potential transmission dynamics across a multi-provincial egg distribution network were discovered by genomic source tracing. In China, multidisciplinary and integrated approaches were coordinated with unprecedented efficiency and scale in this foodborne disease outbreak response, resulting in the prompt intervention of a large, cross-jurisdictional Salmonella outbreak.

In conclusion, in our Research Topic we have provided comprehensive, reliable, and updated information on the current state of knowledge and research in the field of food safety and public health. Food safety remains an important issue affecting individuals' and communities' health and wellbeing. To address this issue, it is important that we take a holistic approach to food safety, addressing not only the immediate concerns of foodborne illness but also the long-term sustainability and health impacts of our food production methods. This includes continuing to pursue research to monitor the epidemiology of food-borne pathogens and ecology AMR in the food supply chain, improving diagnostic methods for the detection and of foodborne pathogens, and innovation of antibiotic-independent approaches for improving food safety. These efforts should be achieved in parallel to increase transparency in the food production process, strengthen regulations, and promote sustainable and organic farming practices. Additionally, consumers should also take steps to ensure the safety of our food by being mindful of where our food comes from and how it is produced. Overall, food safety is a complex issue that requires one health-based multifaceted approach to ensure the health and wellbeing of individuals, communities, and the surrounding environment. By working together, we can take steps to ensure that the food we eat is safe, nutritious, and sustainable for all.

## Author contributions

All authors listed have made a substantial, direct, and intellectual contribution to the work and approved it for publication.

